# EF24 Suppresses Invasion and Migration of Hepatocellular Carcinoma Cells* In Vitro* via Inhibiting the Phosphorylation of Src

**DOI:** 10.1155/2016/8569684

**Published:** 2016-11-23

**Authors:** Ran Zhao, Lamtin Tin, Yuhua Zhang, Yiqi Wu, Yinji Jin, Xiaoming Jin, Fengmin Zhang, Xiaobo Li

**Affiliations:** ^1^Department of Pathology, Harbin Medical University, Harbin, Heilongjiang 150081, China; ^2^Department of Hepatopancreatobiliary Surgery, The Affiliated Hospital of Qingdao University, Qingdao, Shandong 266071, China; ^3^Department of Microbiology, Harbin Medical University, Harbin, Heilongjiang 150081, China; ^4^Translation Medicine Center of Northern China, Harbin Medical University, Harbin, Heilongjiang 150081, China; ^5^Basic Medical Institute, Heilongjiang Medical Science Academy, Heilongjiang 150081, China

## Abstract

Diphenyl difluoroketone (EF24), a curcumin analog, is a promising anticancer compound that exerts its effects by inhibiting cell proliferation and inducing apoptosis. However, the efficacy of EF24 against cancer metastasis, particularly in hepatocellular carcinoma (HCC), remains elusive. In this study, the effect of EF24 on HCCLM-3 and HepG2 cell migration and invasion was detected by wound healing and transwell assay, respectively. The results revealed that EF24 suppressed the migration and invasion of both HCCLM-3 and HepG2 cells. Furthermore, EF24 treatment decreased the formation of filopodia on the cell surface and inhibited the phosphorylation of Src in both cell lines, which may help contribute towards understanding the mechanism underlying the suppressive effect of EF24 on HCC migration and invasion. Additionally, the expression of total- and phosphorylated-Src in primary HCC tissues and their paired lymph node metastatic tissues was detected, and phosphorylated-Src was found to be associated with HCC lymph node metastasis. The results of this study suggest that Src is a novel and promising therapeutic target in HCC and provide evidence to support the hypothesis that EF24 may be a useful therapeutic agent for the treatment of HCC.

## 1. Introduction

Hepatocellular carcinoma (HCC) is one of the most aggressive malignancies and the third leading cause of cancer-associated mortality worldwide [1]. The survival rate for the majority of patients is poor owing to the high incidence of postoperative recurrence and metastasis [2, 3]. Therefore, the development of an effective therapy to impede HCC metastasis remains a challenge.

Curcumin, a constituent of turmeric powder derived from the rhizome of* Curcuma longa*, is well known for its promising antiproliferative activity in many human cancers [4, 5]. It inhibits the enhancer of zeste homologue 2, signal transducer and activator of transcription 3, macrophage stimulating 1, and nuclear factor-*κ*B signaling pathways that are critical in cancer development and progression [6–9]. However, natural curcumin has limited uses due to its poor absorption and low bioavailability [10].

Diphenyl difluoroketone (EF24), an artificially designed structural analog of curcumin, has been shown to be an effective and promising anticancer agent [11]. EF24 exerts anticancer effects via inhibition of cancer growth and induction of cancer cell apoptosis. It has been reported that EF24 induces G2/M arrest and apoptosis by increasing phosphatase and tensin homologue expression in ovarian cancer cells [12]. In addition, it has been reported to decrease lung cancer cell viability by increasing the rate of phosphorylation of extracellular signal-regulated kinase, c-Jun N-terminal kinase, and p38 [13] and promoting apoptosis in HCC [14]. Recently, EF24 has been shown to suppress epithelial-to-mesenchymal transition (EMT) in melanoma cells by upregulating the expression of microRNA- (miR-) 33b [15], which implies that EF24 may inhibit cancer metastasis. However, limited information is available regarding the effect of EF24 on cancer metastasis, particularly in HCC.

In the present study, we examined the effect of EF24 on the migration and invasion of HCC cells. Additionally, we compared the expression of Src in HCC tissues with that in their paired lymph node metastasized tissues and evaluated the effect of EF24 on Src expression in HCC cells.

## 2. Materials and Methods

### 2.1. Reagents and Cell Culture

EF24 was purchased from Sigma (St. Louis, MO, USA). The HCCLM-3 cell line with high metastatic potential, derived from MHCC97 parental cells [16], was provided by the Liver Cancer Institute, Shanghai Medical College of Fudan University (Shanghai, China). HCCLM-3 and HepG2 cells were cultured in Dulbecco's modified Eagle's medium (HyClone; Logan, UT, USA) supplemented with 10% fetal bovine serum (FBS; Gibco; Carlsbad, CA, USA) and 1% antibiotic (100 IU/mL penicillin and 100 *μ*g/mL streptomycin; Mediatech, Inc., Manassas, VA, USA). Cells were incubated in 5% CO_2_ at 37°C.

### 2.2. Cell Viability and Cell Apoptosis Assays

Cell viability was detected by using an MTT assay, as previously described [17]. Briefly, HCCLM-3 and HepG2 cells were seeded into 96-well plates at 5 × 10^3^ cells/well and incubated overnight at 37°C. After treatment with EF24 at different doses (0–8 *μ*M) for 12 h, cells were incubated with 20 *μ*L MTT (5 mg/mL) for 4 h. Then, the culture medium was removed and 150 *μ*L dimethyl sulfoxide (DMSO) was added. The absorbance of each well was read at 490 nm using a microplate reader (model 680; Bio-Rad Laboratories, Inc., Hercules, CA, USA). Cell viability was expressed as the percentage of absorbance of treated wells compared with that of untreated wells (DMSO control). Values (mean ± SD) are from five independent experiments.

A phycoerythrin- (PE-) labeled annexin V apoptosis detection kit (BD Biosciences) was used to detect apoptosis, according to the manufacturer's instructions. Briefly, HCCLM-3 and HepG2 cells (1 × 10^6^ cells) were exposed to 1 *μ*M EF24 for 12 h. Then, the cells were collected, washed, and resuspended in binding buffer at 1 × 10^6^ cells/mL, and then 1 × 10^5^ cells were incubated with 5 *μ*L PE-annexin V and 5 *μ*L 7-aminoactinomycin D for 15 min at room temperature in the dark. Finally, apoptotic cells were analyzed using a flow cytometer (BD Biosciences). Experiments were repeated twice.

### 2.3. Detection of Cellular Ultrastructure Alteration with Electron Microscopy

Electron microscopy was employed to detect cellular ultrastructure changes after treatment with EF24, as previously described [17]. Briefly, HCCLM-3 and HepG2 cells, with or without EF24 treatment, were collected and fixed in 2.5% glutaraldehyde overnight. Then, the cells were fixed with 1% osmium tetroxide for 1 h, dehydrated in a graded series of acetone, and embedded in Epon-812 (Nacalai Tesque, Inc., Osaka, Japan). Ultrathin sections were cut, double-stained with uranyl acetate and lead citrate, and examined under a JEM-1220 electron microscope (JEOL, Ltd., Tokyo, Japan).

### 2.4. Wound Healing Cell Migration Assay

Equal numbers of HCCLM-3 and HepG2 cells were seeded into 6-well plates one day before treatment with EF24. When the cell confluence reached ~90%, cells were treated with different doses of EF24 for 12 h. Then, an artificial wound was created by using a 200 *μ*L pipette tip and the plates were washed with phosphate-buffered saline to remove the debris. A random field was chosen and photographed at 0 and 24 h. From this, the wound width was measured and the healing ability was represented as a ratio of the 24 h width to 0 h width from the same field. The experiments were performed in triplicate.

### 2.5. Transwell Cell Invasion Assay

After treatment with EF24 for 12 h, cells were trypsinized for the Matrigel invasion assay. Matrigel-coated (BD Biosciences) transwell chambers (Corning Costar, Corning, NY, USA) were used to detect the invasion of HCCLM-3 and HepG2 cells, as previously described [17]. Briefly, filters were precoated with 30 *μ*L Matrigel for 3 h, and 4 × 10^4^ cells in serum-free medium were added to the upper chambers. The lower chambers were supplemented with medium containing 10% FBS. After incubation at 37°C for 24 h, the invaded cells were fixed, stained with hematoxylin and eosin, counted, and photographed under a light microscope. Experiments were conducted in triplicate and cell numbers were expressed as mean ± SD.

### 2.6. Western Blotting

EF24-treated and EF24-untreated HCCLM-3 and HepG2 cells were collected for Western blotting analysis, as described in our previous report [18]. Total protein was extracted, and samples were separated by 12% SDS-PAGE and transferred to polyvinylidene difluoride membranes (Merck Millipore). The membranes were incubated with primary antibodies against total- (t-) Src (cat. number 2109; Cell Signaling Technology, Inc., Danvers, MA, USA) and phosphorylated- (p-) Y416Src (cat. number 2101; Cell Signaling Technology, Inc.) at 1 : 1,000 dilution overnight at 4°C, washed in TBST, and then exposed to alkaline phosphatase-conjugated secondary antibody (1 : 800 dilution; Santa Cruz Biotechnology, Inc., Danvers, MA, USA) for 2 h at room temperature. Final detection was performed using Western blue (Promega, Madison, WI, USA). GAPDH was used as an internal control. The blots were imaged and the densitometric readings for the proteins were normalized to those of GAPDH (Quantity One software, version 4.4.0.36; Bio-Rad Laboratories, Inc.).

### 2.7. Immunocytochemistry

HCCLM-3 and HepG2 cells, with or without EF24 treatment, were fixed in 95% ethanol and permeabilized in 0.2% Triton X-100 (Sigma-Aldrich). Then, 3% H_2_O_2_ was used to arrest endogenous peroxidase activity. The standard indirect horseradish peroxidase method was used for staining of the cells. Briefly, cell slides were incubated with antibodies against t-Src (1 : 400 dilution) and p-Y416Src (1 : 50 dilution) overnight at 4°C and then incubated with secondary antibody (PV-6001; ZSGB-Bio, Beijing, China) for 1 h at 37°C. After incubation with 3,3′-diaminobenzidine substrate (ZLI-9019; ZSGB-Bio) for 30 s, the cell slides were counterstained with hematoxylin, dehydrated, and mounted.

### 2.8. Immunohistochemistry

A total of six metastatic lymph node tissue samples and their six primary HCC tissue samples were obtained from Chinese patients diagnosed as having HCC. Sample collection was approved by the Harbin Medical University (Harbin, China) Institutional Ethics Committee. Formalin-fixed paraffin-embedded sections were incubated with t-Src and p-Y416Src antibodies, as described previously [19]. Briefly, the sections were heated for antigen retrieval at 95°C and blocked with 10% goat serum for 1 h. The slides were stained using the standard indirect horseradish peroxidase method, as described above for immunocytochemistry.

Src expression in HCC and lymph node tissues was assessed using the histoscore method, developed by Allred et al. [20]. In each specimen, the overall Src expression was calculated as a sum of the intensity (0, none; 1, weak; 2, moderate; and 3, strong) and proportion (0, none; 1, <5%; 2, 5–25%; 3, 26–50%; 4, 51–75%; and 5, >75%) scores to give a range of 0–8. Three investigators scored the slides independently and an agreement was reached for all samples.

### 2.9. Statistical Analysis

Statistical analyses were performed using SPSS software (version 10.0; SPSS, Inc., Chicago, IL, USA). All data were expressed as the mean ± SD. Comparisons between two groups were analyzed using Student's *t*-test. Mann–Whitney *U* test was used to analyze the differences in Src expression between groups. *P* < 0.05 was considered to indicate a statistically significant difference.

## 3. Results

### 3.1. EF24 Inhibits HCC Cell Invasion and Migration without Affecting Cell Growth and Apoptosis

**Figure 1:**
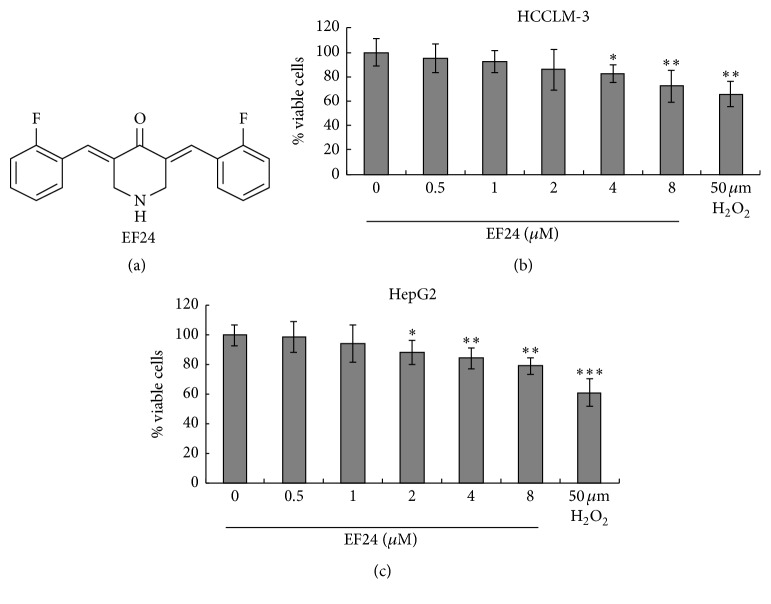
EF24 inhibits HCC cell viability in a dose-dependent manner. (a) The compound structure of EF24. (b) EF24 treatment inhibits the cell viability of HCCLM-3 cells. (c) EF24 treatment inhibits the cell viability of HepG2 cells. HCCLM-3 cells and HepG2 cells were treated with different doses (0–8 *μ*M) of EF24 or 50 *μ*M H_2_O_2_ (positive control) for 12 h; the cell viability was tested by MTT and expressed as the percentage of absorbance of treated wells compared with that of untreated wells. Data were shown as mean ± SD from five repeated experiments. ^*∗*^ *P* < 0.05, ^*∗∗*^ *P* < 0.01, and ^*∗∗∗*^ *P* < 0.001.

**Figure 2:**
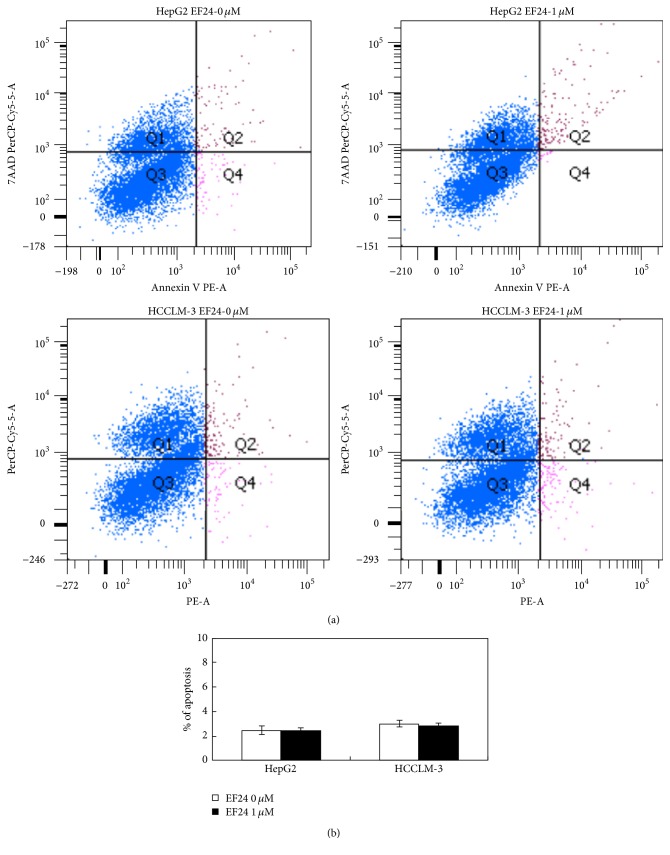
1 *μ*M EF24 treatment does not induce cell apoptosis in HCCLM-3 and HepG2 cell. (a) The representative flow cytometry result to show the cell apoptosis in two HCC cell lines treated with 0 *μ*M and 1 *μ*M EF24 for 12 h. (b) The statistical analysis about the apoptosis of HepG2 and HCCLM-3 cells. There is no significant difference for apoptosis in both cell lines with or without EF24 treatment.

Before evaluating the effects of EF24 on HCC cell invasion and migration* in vitro*, we first determined the concentrations of EF24 that could be used for subsequent cell treatment. HCCLM-3 and HepG2 cells were treated with different concentrations of EF24 (0, 0.5, 1, 2, 4, and 8 *μ*M) for 12 h, and then the viability and apoptosis of both cell lines were determined by MTT assay and flow cytometry, respectively. Our results showed that EF24 inhibited HCCLM-3 and HepG2 cell proliferation in a dose-dependent manner. EF24 at a concentration of <2 *μ*M did not suppress HCCLM-3 cell viability (*P* = 0.508, *P* = 0.293, and *P* = 0.167; Figure 1) and, at concentration <1 *μ*M, did not significantly decrease the viability of HepG2 cells (*P* = 0.367, *P* = 0.407; Figure 1). In addition, 1 *μ*M EF24 treatment did not affect the apoptosis rate in both cell lines (Figure 2). Therefore, 0.5 and 1 *μ*M EF24 were used to treat both cell lines to evaluate the anti-invasion and anti-migration effect of EF24, since these doses have no significant effects on the proliferation and apoptosis of HCC cells.

**Figure 3:**
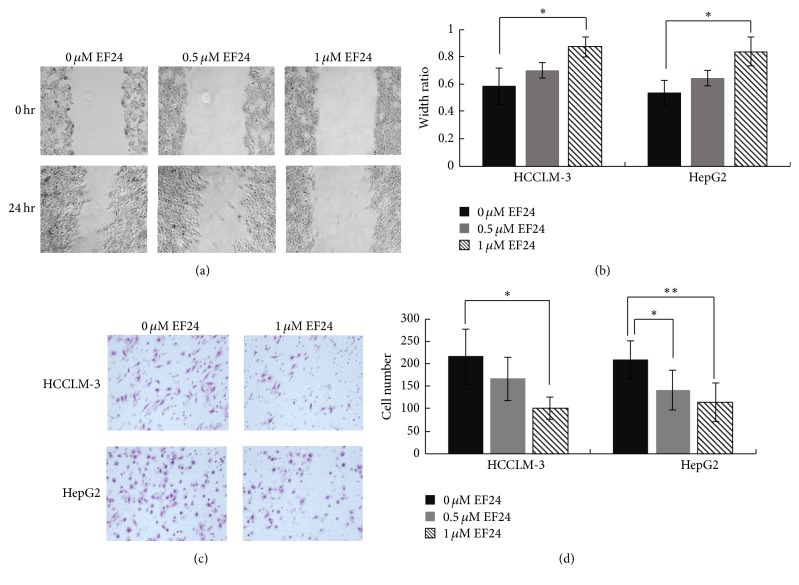
EF24 inhibits the migration and invasion of HCC. (a) EF24 treatment inhibits the migration of HCCLM-3 and HepG2 cells. The artificial wound was created by using a 200 *μ*L pipette tip, and then a random field was chosen and photographed at 0 and 24 h, respectively. Representative images at 0 h and 24 h after wounding were shown at magnification of 100x. (b) Statistical analysis about the effect of EF24 on the migration of HCC. The wound width was measured and the healing ability was represented as a ratio of the 24 h width to 0 h width from the same field. (c) EF24 treatment inhibits the invasion of HCCLM-3 and HepG2 cells. After treatment with or without EF24 for 24 h, cell invasion ability was detected by transwell assay. The invaded cells were fixed, stained, and photographed under a light microscope. (d) Statistical analysis about the effect of EF24 on the invasion of HCC. Data are expressed as the average number of invaded cells under high power field from triplicate experiments. ^*∗*^ *P* < 0.05; ^*∗∗*^ *P* < 0.01.

Transwell and wound healing assays were performed to determine the effect of EF24 on HCC cell invasion and migration, respectively. HCCLM-3 and HepG2 cells showed a dose-dependent decrease in wound healing activities after EF24 treatment; EF24 at 1 *μ*M significantly inhibited HCC migration (*P* = 0.047, *P* = 0.022; Figures 3(a) and 3(b)). Additionally, the transwell assay showed that the invasion ability of HCCLM-3 and HepG2 cells was reduced by EF24 treatment in a dose-dependent manner (Figures 3(c) and 3(d)). These results suggest that EF24 has a potent antimetastasis effect on HCCs and that this effect is independent of its antiproliferative and proapoptotic effects.

### 3.2. EF24 Treatment Decreases the Formation of Filopodia on the Surface of HCC Cells

**Figure 4:**
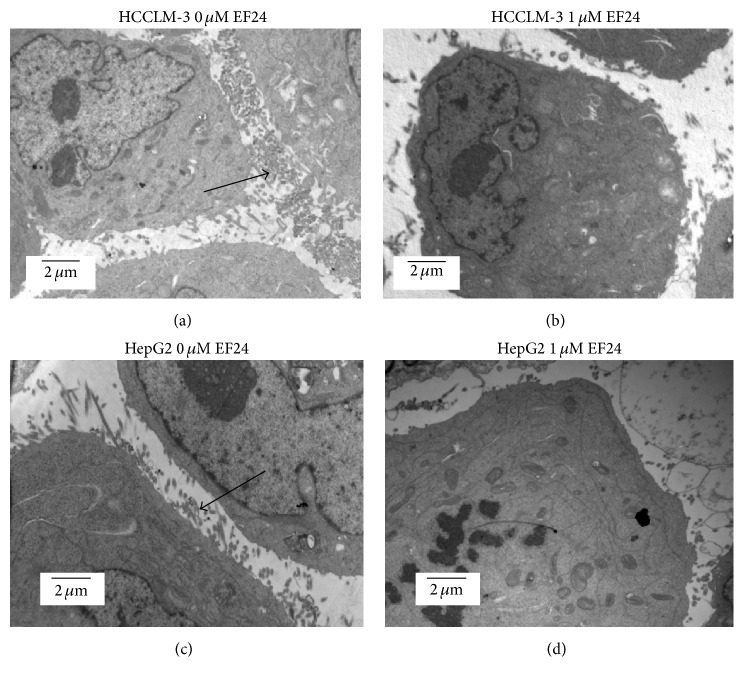
EF24 treatment decreases the formation of filopodia on the surface of HCC. HCCLM-3 and HepG2 cells were incubated with or without EF24 for 12 h and then harvested. Electron microscopy was employed to detect the impact of EF24 on the cellular ultrastructure. (a) and (c) showed that HCCLM-3 and HepG2 cells without EF24 treatment have plentiful filopodia (arrow) (×6000). (b) and (d) showed that the filopodia on the surface of HCCLM-3 and HepG2 cells treated with 1 *μ*M EF24 were decreased (×6000).

To explore how EF24 affects cell migration and invasion, we detected the cellular ultrastructural changes in HCCLM-3 and HepG2 cells after EF24 treatment. HCCLM-3 and HepG2 cells that were not treated with EF24 exhibited abundant organelles, intact nuclei, and plentiful filopodia formation on the cell surface (Figures 4(a) and 4(c)). However, cells treated with 1 *μ*M EF24 for 12 h showed markedly decreased filopodia formation (Figures 4(b) and 4(d)) and organelle degeneration. This result suggests that reduction of filopodia may contribute towards the effect of EF24 in suppressing the invasion and migration of HCC cells.

### 3.3. EF24 Inhibits the Phosphorylation of Src in HCC Cells

**Figure 5:**
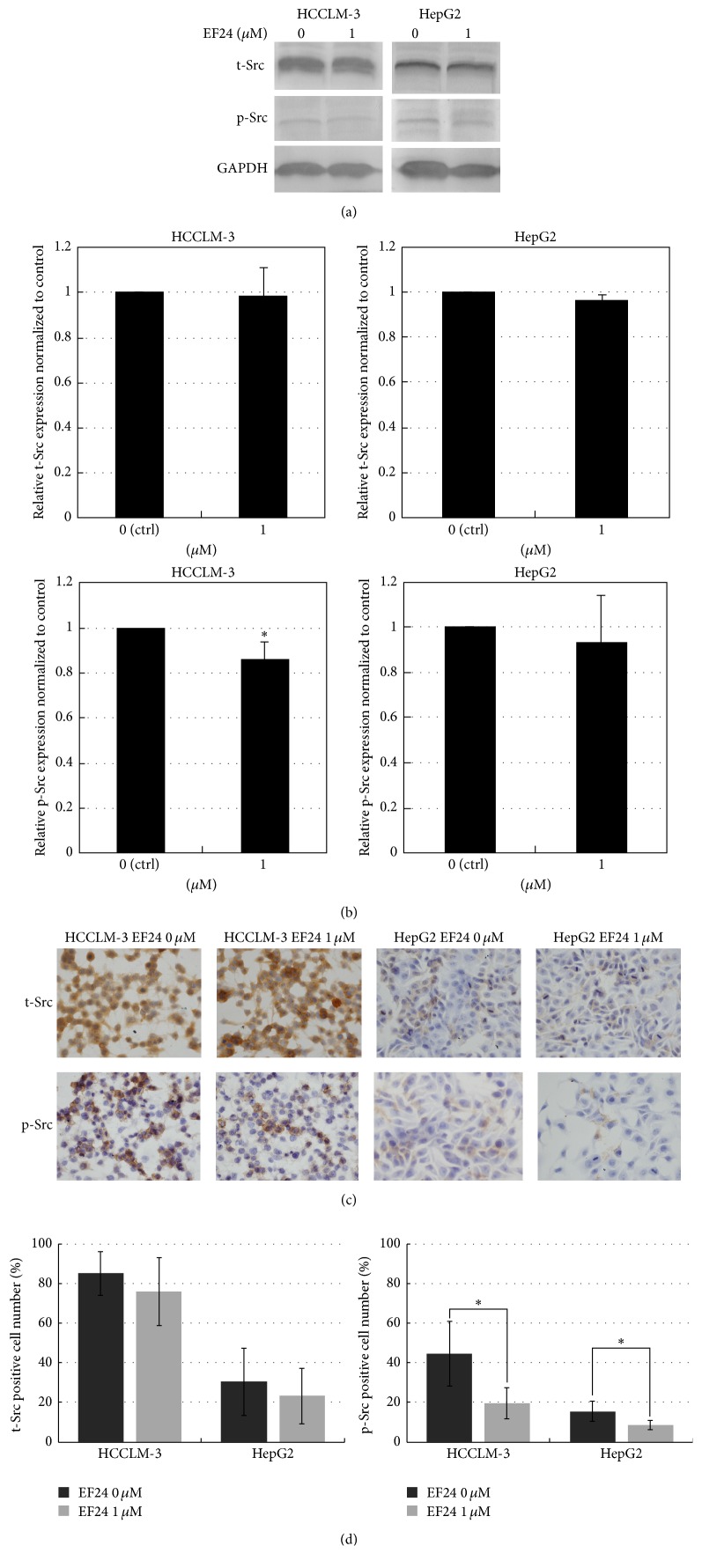
EF24 inhibits the phosphorylation of Src in HCC cells. (a) Detection of the expression of t-Src and p-Src in HCCLM-3 and HepG2 cells treated with 1 *μ*M EF24 by using Western blot assay. (b) Scanning densitometric analysis of Western blot visualizing the relative levels of t-Src and p-Src in HCCLM-3 and HepG2 cells treated with 1 *μ*M EF24. EF24 treatment reduced p-Src but not t-Src level in HCCLM-3 cells. Data were shown as mean ± SD of three independent experiments, ^*∗*^ *P* < 0.05. (c) Detection of the expression of t-Src and p-Src in HCCLM-3 and HepG2 cells treated with 1 *μ*M EF24 by using immunocytochemistry staining (×400). (d) Statistical analysis of the percentage of t-Src or p-Src staining positive cells. EF24 treatment reduced p-Src but not t-Src level in both HCCLM-3 and HepG2 cells. Data were shown as mean ± SD of three independent experiments, ^*∗*^ *P* < 0.05.

Recently, Src has been shown to serve important roles in promoting HCC [21, 22]. Therefore, we detected the effect of EF24 treatment on the expression of Src. The expression of t-Src and p-Y416Src was detected in HepG2 and HCCLM-3 cells following treatment with or without EF24. Our result showed that EF24 treatment (1 *μ*M) attenuated the phosphorylated-Src but did not affect the total-Src level (Figures 5(a) and 5(b)). Consistent with the Western blotting results, immunocytochemistry staining indicated that EF24 treatment decreased the phosphorylation of Src in both HCCLM-3 and HepG2 cells (Figures 5(c) and 5(d)). These results suggest that EF24 suppression on migration and invasion may be attributed to its inhibitory effect on the phosphorylation of Src in HCC cells.

### 3.4. Src Expression Increases in the Metastatic Lymph Node Tissue of HCC Patients

**Figure 6:**
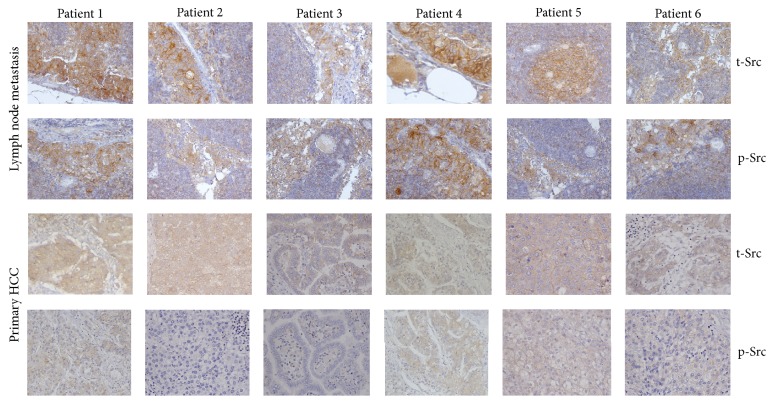
Representative immunohistochemistry results about Src expression in six human primary HCC patients and their paired lymph node metastasis tissues (magnification, ×200).

The expression of t-Src and p-Y416Src was evaluated by immunohistochemistry in human primary HCC samples and their paired lymph node metastasized tissues to detect their potential effects on HCC metastasis (Figure 6). The histoscore was analyzed using Mann–Whitney *U* test and the result revealed that the staining scores of t-Src (5.33 ± 1.21) and p-Y416Src (3.00 ± 1.10) were both significantly higher in metastatic lymph node tissue compared with those in the primary liver HCC tissue (*P* = 0.012 and *P* = 0.030, resp.; Table 1). These results suggest that Src may be a potential target for preventing and treating HCC metastasis.

## 4. Discussion

Curcumin, as a promising anticancer agent, has attracted increasing attention for its antiproliferative and chemopreventive properties [4, 5]. An increasing amount of evidence showed that curcumin inhibits cancer metastasis via different mechanisms. For example, curcumin inhibits miR-21 transcription and suppresses invasion and metastasis in colorectal cancer [23]. In addition, curcumin inhibits breast cancer metastasis by decreasing the inflammatory cytokines, chemokine (C-X-C motif) ligand 1 (CXCL1) and CXCL2 [24]. Furthermore, curcumin was found to reduce the expression of SET8 to inhibit metastasis in pancreatic cancer [25]. EF24, a novel curcumin analog with greater biological activity and bioavailability [26, 27], has been shown to possess antiproliferative ability in anticancer screens [11]. However, few studies have detected the effect of EF24 on cancer metastasis. The present study demonstrates for the first time, to the best of our knowledge, that EF24 inhibits HCC cell migration and invasion.

Cellular migration is a tightly coordinated mechanism essential in physiological processes and cancer invasion and metastasis [28]. Filopodia are thin, finger-like, actin-rich membrane protrusions [29] that command the direction of the migrating cells and contribute towards cancer cell invasion [30, 31]. Notably, in this study, EF24 was found to reduce the migratory and invasion potential of HCC cells and reduce the quantity of filopodia present. This result can be supported by a previous study that demonstrated that curcumin targets breast cancer stem-like cells with microtentacles as an antimetastatic strategy [32]. In addition, a previous study observed that EF24 disrupts the microtubule cytoskeleton and inhibits hypoxia-inducible factor-1 [33]. Therefore, it is reasonable that the reduction of filopodia may contribute to the lower migration ability under EF24 treatment in HCC cells.

Src, a nonreceptor tyrosine kinase, is a critical modulator of multiple signaling pathways mediated by integrin-extracellular matrix interactions [34]. Activated Src (p-Y416Src) initiates signaling pathways that induce cell proliferation, migration, and invasion [35]. Additionally, we previously found that high Src expression scores in HCC tissues were associated with positive lymph node metastasis status [36]. In this study, we further investigated the status of activated Src in primary HCC tissues and their paired lymph nodes with HCC metastasis. The results demonstrated that p-Y416Src is expressed significantly higher in metastatic lymph node tissue compared with that in paired primary liver HCC tissues. Curcumin was reported to regulate the expression of the Src-Akt axis via modulation of miR-203 in bladder cancer [37]. EF24 was found to inhibit migration and EMT in melanoma cells via the suppression of Src and high-mobility group AT-hook 2 [15]. Thus, in this study, we determined whether EF24 inhibits HCC metastasis by downregulating the expression or activation of Src. Then, we examined the expression and phosphorylation change of Src in HCC cells following treatment with EF24 by using Western blot and immunocytochemistry assay analysis. As expected, we demonstrated that treatment with EF24 inhibits the phosphorylation of Src instead of affecting the total level of the protein. This result is consistent with a previous study [15]. These data suggest that Src may be a potential target for HCC metastasis and that inhibition of the phosphorylation of Src may be a molecular mechanism underlying EF24 inhibiting the metastasis of HCC cells.

## 5. Conclusion

In summary, we demonstrated that EF24 suppresses HCC migration and invasion* in vitro*. This study provides evidence to support that EF24 may be a useful therapeutic reagent for the treatment of hepatocellular carcinoma and suggests that Src is a novel and promising therapeutic target in hepatocellular carcinoma.

## Figures and Tables

**Table 1:** Src expression in metastatic lymph nodes and liver primary lesions of HCC (*n* = 6).

Variable	Metastatic lymph nodes	Liver primary lesions	*P*
t-Src	5.33 ± 1.21	3.17 ± 0.98	0.012
p-Y416Src	3.00 ± 1.10	1.33 ± 1.03	0.030

Mean histoscore values ± SD were calculated for t-Src and p-Y416Src expression in lymph node metastasis and liver primary lesions of HCC.

## References

[1] Lin S., Hoffmann K., Schemmer P. (2012). Treatment of hepatocellular carcinoma: a systematic review. *Liver Cancer*.

[2] Tang Z.-Y. (2001). Hepatocellular carcinoma-cause, treatment and metastasis. *World Journal of Gastroenterology*.

[3] Tang Z., Zhou X., Lin Z. (1999). Surgical treatment of hepatocellular carcinoma and related basic research with special reference to recurrence and metastasis. *Chinese Medical Journal*.

[4] Sahu R. P., Batra S., Srivastava S. K. (2009). Activation of ATM/Chk1 by curcumin causes cell cycle arrest and apoptosis in human pancreatic cancer cells. *British Journal of Cancer*.

[5] Wahl H., Tan L., Griffith K., Choi M., Liu J. R. (2007). Curcumin enhances Apo2L/TRAIL-induced apoptosis in chemoresistant ovarian cancer cells. *Gynecologic Oncology*.

[6] Bao B., Ali S., Banerjee S. (2012). Curcumin analogue CDF inhibits pancreatic tumor growth by switching on suppressor microRNAs and attenuating EZH2 expression. *Cancer Research*.

[7] Seo J. H., Jeong K. J., Oh W. J. (2010). Lysophosphatidic acid induces STAT3 phosphorylation and ovarian cancer cell motility: their inhibition by curcumin. *Cancer Letters*.

[8] Yu T., Ji J., Guo Y.-L. (2013). MST1 activation by curcumin mediates JNK activation, Foxo3a nuclear translocation and apoptosis in melanoma cells. *Biochemical and Biophysical Research Communications*.

[9] Kasinski A. L., Du Y., Thomas S. L. (2008). Inhibition of I*κ*B kinase-nuclear factor-*κ*B signaling pathway by 3,5-bis(2-flurobenzylidene)piperidin-4-one (EF24), a novel monoketone analog of curcumin?. *Molecular Pharmacology*.

[10] Anand P., Kunnumakkara A. B., Newman R. A., Aggarwal B. B. (2007). Bioavailability of curcumin: problems and promises. *Molecular Pharmaceutics*.

[11] Adams B. K., Cai J., Armstrong J. (2005). EF24, a novel synthetic curcumin analog, induces apoptosis in cancer cells via a redox-dependent mechanism. *Anti-Cancer Drugs*.

[12] Selvendiran K., Tong L., Vishwanath S. (2007). EF24 induces G2/M arrest and apoptosis in cisplatin-resistant human ovarian cancer cells by increasing PTEN expression. *The Journal of Biological Chemistry*.

[13] Thomas S. L., Zhao J., Li Z. (2010). Activation of the p38 pathway by a novel monoketone curcumin analog, EF24, suggests a potential combination strategy. *Biochemical Pharmacology*.

[14] Liang Y., Yin D., Hou L. (2011). Diphenyl difluoroketone: a potent chemotherapy candidate for human hepatocellular carcinoma. *PLoS ONE*.

[15] Zhang P., Bai H., Liu G. (2015). MicroRNA-33b, upregulated by EF24, a curcumin analog, suppresses the epithelial-to-mesenchymal transition (EMT) and migratory potential of melanoma cells by targeting HMGA2. *Toxicology Letters*.

[16] Li Y., Tang Z. Y., Ye S. L. (2003). Establishment of a hepatocellular carcinoma cell line with unique metastatic characteristics through in vivo selection and screening for metastasis-related genes through cDNA microarray. *Journal of Cancer Research and Clinical Oncology*.

[17] Zhao R., Wang T.-Z., Kong D. (2011). Hepatoma cell line HepG2.2.15 demonstrates distinct biological features compared with parental HepG2. *World Journal of Gastroenterology*.

[18] Wang T., Zhao R., Wu Y. (2011). Hepatitis B virus induces G1 phase arrest by regulating cell cycle genes in HepG2.2.15 cells. *Virology Journal*.

[19] Wu Y., Wang T., Ye S. (2012). Detection of hepatitis B virus DNA in paraffin-embedded intrahepatic and extrahepatic cholangiocarcinoma tissue in the northern Chinese population. *Human Pathology*.

[20] Allred D. C., Clark G. M., Elledge R. (1993). Association of p53 protein expression with tumor cell proliferation rate and clinical outcome in node-negative breast cancer. *Journal of the National Cancer Institute*.

[21] Masaki T., Okada M., Shiratori Y. (1998). pp60(c-src) activation in hepatocellular carcinoma of humans and LEC rats. *Hepatology*.

[22] Sun C. K., Man K., Ng K. T. (2008). Proline-rich tyrosine kinase 2 (Pyk2) promotes proliferation and invasiveness of hepatocellular carcinoma cells through c-Src/ERK activation. *Carcinogenesis*.

[23] Mudduluru G., George-William J. N., Muppala S. (2011). Curcumin regulates miR-21 expression and inhibits invasion and metastasis in colorectal cancer. *Bioscience Reports*.

[24] Kronski E., Fiori M. E., Barbieri O. (2014). MiR181b is induced by the chemopreventive polyphenol curcumin and inhibits breast cancer metastasis via down-regulation of the inflammatory cytokines CXCL1 and -2. *Molecular Oncology*.

[25] Ma J., Fang B., Zeng F. (2014). Curcumin inhibits cell growth and invasion through up-regulation of miR-7 in pancreatic cancer cells. *Toxicology Letters*.

[26] Adams B. K., Ferstl E. M., Davis M. C. (2004). Synthesis and biological evaluation of novel curcumin analogs as anti-cancer and anti-angiogenesis agents. *Bioorganic and Medicinal Chemistry*.

[27] Reid J. M., Buhrow S. A., Gilbert J. A. (2014). Mouse pharmacokinetics and metabolism of the curcumin analog, 4-piperidinone,3,5-bis[(2-fluorophenyl)methylene]-acetate(3E,5E) (EF-24; NSC 716993). *Cancer Chemotherapy and Pharmacology*.

[28] Condeelis J., Segall J. E. (2003). Intravital imaging of cell movement in tumours. *Nature Reviews Cancer*.

[29] Suraneni P., Rubinstein B., Unruh J. R., Durnin M., Hanein D., Li R. (2012). The Arp2/3 complex is required for lamellipodia extension and directional fibroblast cell migration. *The Journal of Cell Biology*.

[30] Bryce N. S., Clark E. S., Leysath J. L., Currie J. D., Webb D. J., Weaver A. M. (2005). Cortactin promotes cell motility by enhancing lamellipodial persistence. *Current Biology*.

[31] Stevenson R. P., Veltman D., Machesky L. M. (2012). Actin-bundling proteins in cancer progression at a glance. *Journal of Cell Science*.

[32] Charpentier M. S., Whipple R. A., Vitolo M. I. (2014). Curcumin targets breast cancer stem-like cells with microtentacles that persist in mammospheres and promote reattachment. *Cancer Research*.

[33] Thomas S. L., Zhong D., Zhou W. (2008). EF24, a novel curcumin analog, disrupts the microtubule cytoskeleton and inhibits HIF-1. *Cell Cycle*.

[34] Bolós V., Gasent J. M., López-Tarruella S., Grande E. (2010). The dual kinase complex FAK-src as a promising therapeutic target in cancer. *OncoTargets and Therapy*.

[35] Frame M. C. (2002). Src in cancer: deregulation and consequences for cell behaviour. *Biochimica et Biophysica Acta*.

[36] Zhao R., Wu Y., Wang T. (2015). Elevated Src expression associated with hepatocellular carcinoma metastasis in northern Chinese patients. *Oncology Letters*.

[37] Saini S., Arora S., Majid S. (2011). Curcumin modulates microRNA-203-mediated regulation of the Src-Akt axis in bladder cancer. *Cancer Prevention Research*.

