# Corrigendum to “EF24 Suppresses Invasion and Migration of Hepatocellular Carcinoma Cells *In Vitro* via Inhibiting the Phosphorylation of Src”

**DOI:** 10.1155/2021/9839618

**Published:** 2021-06-09

**Authors:** Ran Zhao, Lamtin Tin, Yuhua Zhang, Yiqi Wu, Yinji Jin, Xiaoming Jin, Fengmin Zhang, Xiaobo Li

**Affiliations:** ^1^Department of Pathology, Harbin Medical University, Harbin, Heilongjiang 150081, China; ^2^Department of Hepatopancreatobiliary Surgery, The Affiliated Hospital of Qingdao University, Qingdao, Shandong 266071, China; ^3^Department of Microbiology, Harbin Medical University, Harbin, Heilongjiang 150081, China; ^4^Translation Medicine Center of Northern China, Harbin Medical University, Harbin, Heilongjiang 150081, China; ^5^Basic Medical Institute, Heilongjiang Medical Science Academy, Heilongjiang 150081, China

In the article titled “EF24 Suppresses Invasion and Migration of Hepatocellular Carcinoma Cells In Vitro via Inhibiting the Phosphorylation of Src” [1], errors were identified in Figures 3 and 6.

In Figure 3(a), the incorrect image was included for 0 *μ*M EF24 (0 hr) due to an error in the selection of images during the preparation of the manuscript. The image in the original publication was an alternative image taken at 0.5 *μ*M EF24 (0 hr).

In Figure 6, the incorrect image was included for Patient 5 (p-Src). The image in the original publication was duplicated with the P-Src image for Patient 2 due to an error in the selection of images during the preparation of the manuscript.

The authors confirm that these errors do not affect the conclusions of the article, and corrected Figures 3 and 6 are as follows:

## Figures and Tables

**Figure 1:**
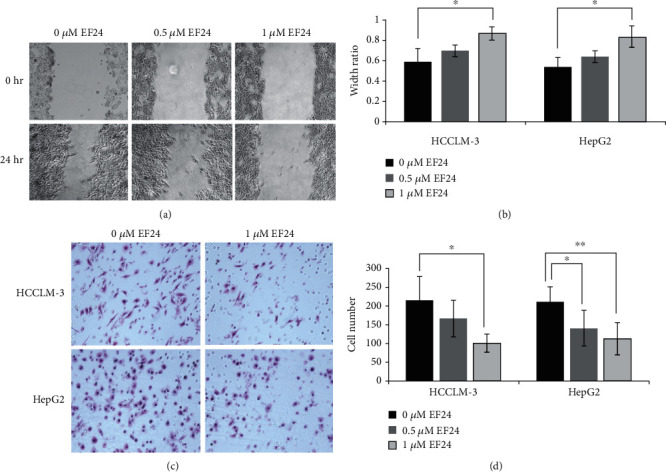
EF24 inhibits the migration and invasion of HCC. (a) EF24 treatment inhibits the migration of HCCLM-3 and HepG2 cells. The artificial wound was created by using a 200 *μ*L pipette tip, and then a random field was chosen and photographed at 0 and 24 h, respectively. Representative images at 0 h and 24 h after wounding are shown at a magnification of 100x. (b) Statistical analysis about the effect of EF24 on the migration of HCC. The wound width was measured, and the healing ability was represented as a ratio of the 24 h width to 0 h width from the same field. (c) EF24 treatment inhibits the invasion of HCCLM-3 and HepG2 cells. After treatment with or without EF24 for 24 h, cell invasion ability was detected by transwell assay. The invaded cells were fixed, stained, and photographed under a light microscope. (d) Statistical analysis about the effect of EF24 on the invasion of HCC. Data are expressed as the average number of invaded cells under high power field from triplicate experiments. ^∗^*P* < 0.05; ^∗∗^*P* < 0.01.

**Figure 2:**
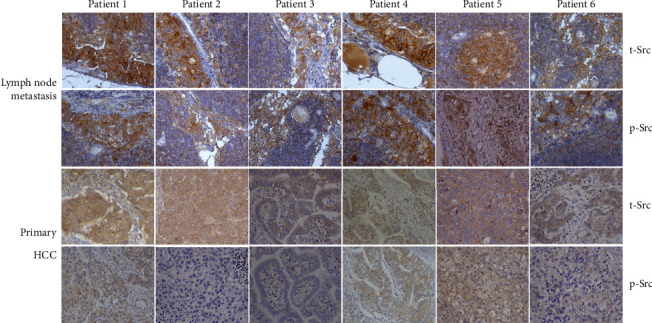
Representative immunohistochemistry results about Src expression in six human primary HCC patients and their paired lymph node metastasis tissues (magnification, ×200).
